# Clinical characteristics and short-term prognosis of LGI1 antibody encephalitis: a retrospective case study

**DOI:** 10.1186/s12883-018-1099-z

**Published:** 2018-07-06

**Authors:** Weishuai Li, Si Wu, Qingping Meng, Xiaotian Zhang, Yang Guo, Lin Cong, Shuyan Cong, Dongming Zheng

**Affiliations:** Department of Neurology, Shengjing Hospital of China Medical University, Sanhao Street 36, Shenyang, 110004 Liaoning China

**Keywords:** Epilepsy, Autoimmune encephalitis, Magnetic resonance imaging, Cognitive function

## Abstract

**Background:**

Recently, most reports of Leucine-rich glioma-inactivated 1 (LGI1) antibody encephalitis are from Europe and the US, while the short term outcome and clinical characteristics of Chinese patients are rarely reported,we study the clinical manifestations, laboratory results and brain magnetic resonance images (MRI) of eight patients who were recently diagnosed with LGI1 antibody encephalitis in our hospital to improve the awareness and knowledge of this disease.

**Methods:**

Eight patients (five males and three females; mean age, 63.4) with LGI1 antibody encephalitis who were diagnosed and treated in the Department of Neurology of Shengjing Hospital of China Medical University from September 2016 to June 2017 were recruited for the current study. Their general information, clinical manifestations, treatment regimens, and short-term prognoses were retrospectively analyzed, as were the results from MRI and laboratory findings.

**Results:**

Overall, patient symptoms included cognitive impairment, which manifested primarily as memory deficits (8/8), seizures (including faciobrachial dystonic seizure, (FBDS)) (8/8), psychiatric and behavioral disorders (7/8), sleep disorders (4/8), and autonomic abnormalities (3/8). Five patients also had abnormal findings on brain MRI, mainly involving the hippocampus, basal ganglia and insula. Hyponatremia occurred in six cases. All patients tested positive for LGI1 antibodies in their serum/cerebrospinal fluid (CSF)and patients were negative for tumors. Symptoms rapidly improved after treatment with immunoglobulin and/or steroid therapy. The patients were followed up for 4–13 months after discharge, and two patients relapsed.

**Conclusion:**

Primary symptoms of LGI1 antibody encephalitis include memory impairments, seizures, FBDS, and mental and behavioral abnormalities. Increased titers of LGI1 antibodies are also present in the serum/CSF of patients. Patients often have hyponatremia, and MRIs show abnormalities in various brain regions. Finally, immunotherapy shows good efficacy and positive benefits, although patients may relapse in the short-term.

## Background

Leucine-rich glioma-inactivated 1 (LGI1) antibody encephalitis is a rare autoimmune voltage-gated potassium channel complex (VGKC) antibody-associated limbic encephalitis. Specifically, it is classified as an antineuronal surface antigen- or antisynaptic protein-associated autoimmune encephalitis [1]. In addition to the common symptoms of limbic encephalitis such as cognitive impairment, seizures, and psychiatric disorders, this disease is also associated with faciobrachial dystonic seizure (FBDS) and refractory hyponatremia [2]. Unlike other limbic encephalitides, LGI1 antibody encephalitis is rarely accompanied by tumors [3] and shows a good response to immunotherapy [4].

Current studies suggest two possible pathogenic mechanisms involving LGI1 antibodies: reducing the formation of the LGI1-ADAM complex and altering the dendritic spine density of neurons located in the dentate gyrus and thalamus [5–9].

In the present study, we summarized and analyzed the clinical manifestations, laboratory results and brain magnetic resonance images (MRI) of eight patients who were recently diagnosed with LGI1 antibody encephalitis in our hospital to improve the awareness and knowledge of this disease.

## Methods

Clinical data from eight patients who were diagnosed with LGI1 antibody encephalitis in the Department of Neurology of Shengjing Hospital of China Medical University from September 2016 to June 2017 were collected and analyzed. Clinical data included the following: clinical manifestations, laboratory and brain MRI results, treatment regimens, follow-up data and prognoses. All patients received a full range of laboratory tests, including standard biochemistry, thyroid function, syphilis, HIV, viral antibodies (including herpes simplex virus 1, 2, and herpes zoster virus), rheumatic indicators, tumor biomarkers and autoimmune encephalitis-related antibodies (NMDAR, LGI1, CASPR2, GABABR, AMPA1R, AMPA2R and other neuronal surface- or synaptic protein-related antibodies and classical paratuberculosis antibodies, such as CV2, Hu, Yo, Ri, Ma, and amphiphysin), as well as other laboratory tests. Some patients also received a cerebrospinal fluid (CSF) test. The blood or CSF autoimmune encephalitis-related antibodies were tested by an indirect immunofluorescence assay using standard kits (Euroimmun Medical Diagnostic (China) Co., Ltd., Beijing, People’s Republic of China). All eight patients underwent brain MRI and dynamic electroencephalography (EEG) examinations; several submitted to a recheck of the titer of the LGI1 antibody during follow-up. This study was approved by the Ethics Committee of Shengjing Hospital in accordance with the Declaration of Helsinki. All participants provided written informed consent documents.

## Results

Eight patients, including five males and three females between 41 and 73 years, participated in the study. The average age of onset of the disease was 63.4 years. The clinical data of all patients are shown in Table 1.

**Table 1:** Clinical manifestations of eight patients with LGI1 antibody encephalitis

Characteristic	Case1	Case2	Case3	Case4	Case5	Case6	Case7	case8
Sex	Male	Male	Female	Female	Male	Male	Female	Male
Age (years)	64	69	60	63	67	73	41	70
Onset to visit (days)	60	45	300	15	20	10	150	30
Initial symptoms	FBDS (50/d)	FBDS(20/d)	Tonic-clonic seizures(12 in total),	Tonic-clonic seizures(5 in total),	Memory deficit,	Memory deficit,	FBDS(100/d)	FBDS(40/d)
Other symptoms	Memory deficit, Partial seizures(arrector pili muscle contraction) Sleep disorder, Autonomic disorders (Sexual dysfunction, sinus bradycardia with chest tightness)	Memory deficit, Tonic-clonic(2 in total), partial seizures(3–5/d) Hallucination(visual+ auditory) Spatial disorientation, Sleep disorder, Ataxia, Autonomic disorders(sweating)	Memory deficit, Hallucinations(auditory) Spatial disorientation, Sleep disorder, Emotional changes (irritability,indifference), Autonomic disorders (Bradycardia)	FBDS(70/d), Memory deficit, Spatial disorientation, Hallucination(visual+ auditory), Emotional changes (indifference)	Partial seizures(10–15/d) Hallucination(visual+ auditory), Sleep disorder	FBDS(50/d) Spatial disorientation Emotional changes (indifference)	Memory deficit, Emotional change (anxiety, irritability suspiciousness)	Memory deficit, Emotional change (anxiety)
MMSE score (0–30)(Education)	University	high school	high school	high school	University	University	University	high school
Admission misse points	26 recall (1) calculation(1) orientation(1) complex commands (1)	16 recall (3) calculation (3) orientation (3)repetition (1) complex commands (4)	18 recall (3) calculation (3) orientation (2) repetition (1) complex commands(3)	15 registration (1) recall (3) calculation (3) orientation (3) repetition (1) complex commands(4)	20 recall (2) calculation (2) orientation (3) complex commands(3)	22 recall (2) calculation (1) orientation (1) complex commands(4)	23 recall (2) calculation (2) orientation (1) complex commands(2)	14 registration (1) recall (3) calculation (3) orientation (3) repetition (1) complex commands(5)
Discharge misse points	30	23 recall (2) calculation (1) orientation (1) complex commands (3)	28 recall (1) complex commands(1)	26 recall (1) calculation (1) orientation (1) complex commands(1)	25 recall (1) calculation (1) orientation (1) complex commands(2)	28 recall (1) orientation (1)	29 recall (1)	26 recall (1) calculation (1) orientation (1) complex commands(1)
LGi1 antibody (serum)	1:10 (before admission) 1:32 (after admission)	1:32	1:32	1:10	1:32	1:32	1:100	1:32
LGi1 antibody (CSF)	1:1	–	–	1:1	1:3.2	1:1	–	–
Blood sodium (normal 135–155 mmol/L)	135.1	130	127.9	126	131.4	121.2	132	140
White blood cell (CSF) (normal 0–5 × 106/L)	4	–	–	2	10	2	–	–
Protein(CSF) (normal 0.15–0.45 g/L)	0.25	–	–	0.31	0.46	0.28	–	–
Glucose(CSF)(normal 2.5–4.5 mmol/L)	3.5	–	–	4.07	3.91	4.1	–	–
Brain MRI	Right insula	Norma	Bilateral hippocampus	Bilateral caudate nucleus+ putamen	Right hippocampus	Normal	Right caudate nucleus	Normal
EEG	Normal	Normal	Normal	Diffuse slow wave (4-6 Hz)	Paroxysmal spike-slow wave (right frontal lobe,middle and posterior temporal lobe)	Normal	Normal	Normal
AEDs	Levetiracetam Lamotrigine	Sodium valproate	Sodium valproate	Sodium valproate	Levetiracetam	Levetiracetam	Sodium valproate	Sodium valproate
Immunotherapy	methylprednisolone +Gamma globulin	methylprednisolone	methylprednisolone	methylprednisolone +Gamma globulin	methylprednisolone +Gamma globulin	methylprednisolone + Gamma globulin	methylprednisolone	methylprednisolone
Symptoms at discharge	FBDS disappeared	FBDS reduction	No seizures	FBDS disappeared	No seizures	FBDS disappeared	FBDS disappeared	FBDS disappeared
Length of stay(days)	13	15	16	12	21	25	17	15
Follow up time(months)	4	4	7	6	6	13	4	4
serum antibody follow up	1:10	–	1:32	–	–	–	–	–
Relapse(months)	No	No	No	1	No	3	No	No
Remaining symptoms	amnesia	amnesia Spatial disorientation	amnesia Insomnia	amnesia	Insomnia	amnesia Spatial disorientation	No	amnesia
AEDs	No	Sodium valproate	Sodium valproate	Sodium valproate	Levetiracetam	Levetiracetam	Sodium valproate	Sodium valproate
Seizure	No	No	No	No	Occasionally(absence seizures)	No	No	No

‘–’, No test information;

Abbreviations: *CSF* cerebrospinal fluid, *FBDS* faciobrachial dystonic seizure, *MMSE* Mini-Mental State Examination, *LGI1* leucine-rich glioma-inactivated 1, *AEDs* antiepileptic drugs, *EEG* electroencephalography

The first and core symptoms in this group of patients were seizures and cognitive disorders. Seizures included tonic-clonic seizures, partial seizures and FBDS; among them, dystonia-like episodes involving ipsilateral face or limbs (FBDS) were most common, while short-term memory impairment was the most obvious manifestation related to cognitive disorders. Spatial disorientation, hallucinations, and emotional changes were also common. Several patients suffered from sleeping disorders and autonomic dysfunctions, for example, sexual dysfunction, sweating and sinus bradycardia.

**Fig. 1:**
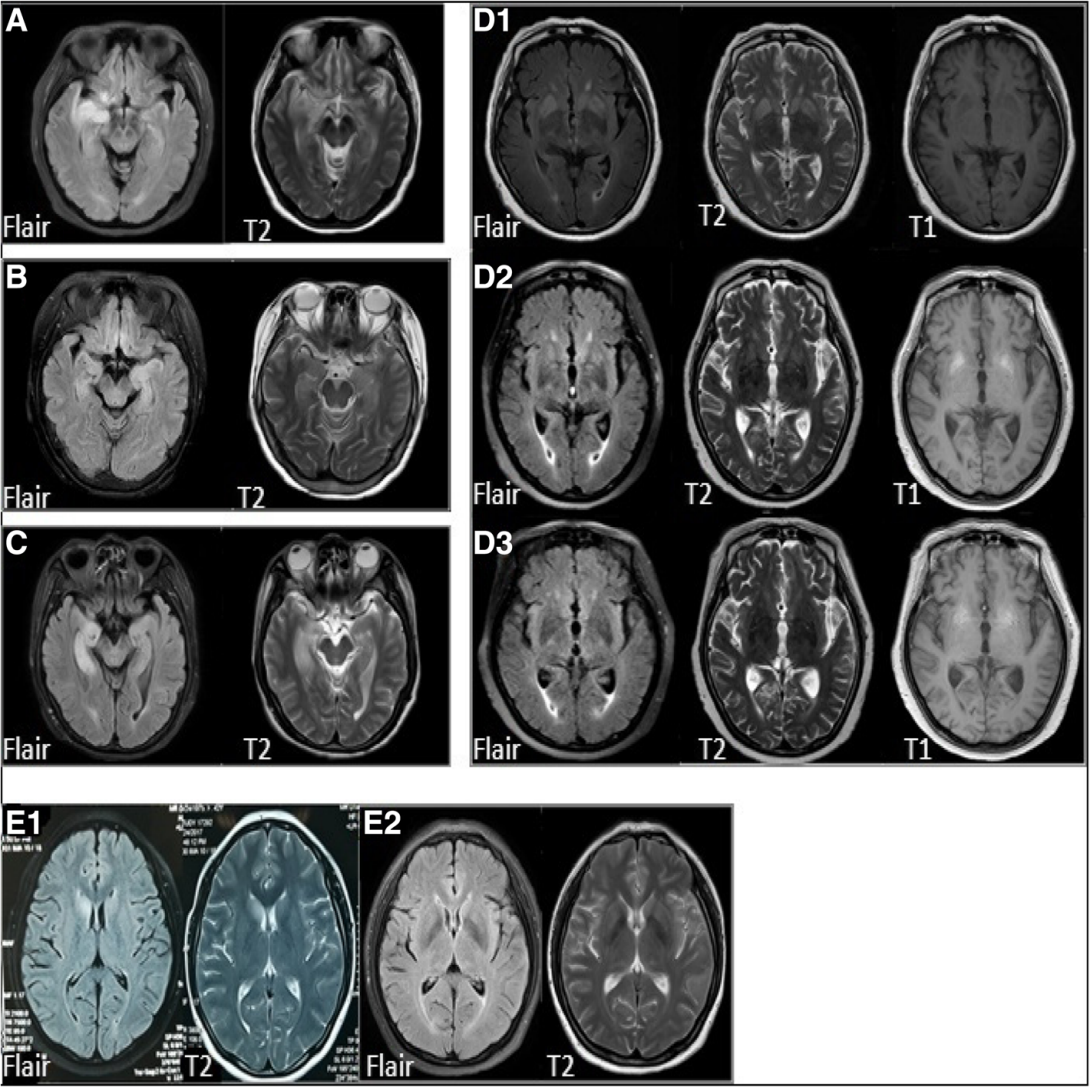
MRI images of five patients with LGI1 antibody encephalitis. **a** FLAIR sequences and high T2 signal changes in the right insula in case 1; **b** FLAIR sequences and high T2 signal changes in the bilateral hippocampus in case 3; **c** FLAIR sequences and high T2 signal changes in the right hippocampus in case 5; **D**1 Abnormal signals in the bilateral caudate nucleus and putamen in case 4 at admission; MRI follow-up after 1 month (**D**2) and 2 months (**D**3) showed that the lesion signal intensity changed to a high T1 signal; (**E**1) Abnormal signals in bilateral caudate nucleus in case 7 at admission; **E**2 MRI of case 7 after 5 months. Abbreviations: MRI, magnetic resonance imaging; FLAIR, fluid-attenuated inversion recovery

Routine CSF examination did not show abnormalities apart from a slightly increased white blood cell count and protein in one patient. All patients were positive for the LGI1 antibody in CSF, although the antibody titer was significantly lower than that in the peripheral blood; all patients tested positive for the LGI1 antibody in their blood. Blood sodium levels in six patients were below normal, and three others had refractory hyponatremia. All patients were negative for other autoimmune encephalitis antibodies. There were no abnormal results for thyroid function, rheumatism series and tumor biomarkers. Two patients had abnormal EEG signals, which presented separately as diffuse slow waves and paroxysmal spike-slow waves. No abnormal EEG signals were detected during FDBS. MRI identified abnormalities (high T2 signaling, low T1 signaling, and high fluid-attenuated inversion recovery [FLAIR] sequences) in the insula, hippocampus, and basal ganglia of five patients (Fig. 1). In one patient, the low T1 signal gradually increased during the follow-up period.

All patients received treatment with oral antiepileptic drugs (AEDs) and glucocorticoid therapy (intravenous infusion of methylprednisolone (1000,500,250,120 mg/d for 3 days each)). Four patients were also given intravenous immunoglobulin (0.4 g/kg/d for 5 days). The average length of stay was 16.8 days, and patients had significantly improved at discharge. All patients continued to take oral prednisone (prednisone tablets from 60 mg/d, decreased by 5 mg every 2 weeks) and AEDs, with a follow-up period of 4–13 months. Two patients relapsed within 3 months, but symptoms improved remarkably after immunotherapy. Primary posttreatment symptoms included mild memory impairments, spatial disorientation, and sleep disorders. Only one patient had subsequent seizures.

## Discussion

Recently, encephalitis cases in which the antibodies target cell-surface or synaptic proteins are being identified with increasing frequency; the antigens include the N-methyl-D-aspartate receptor (NMDAR), the α-amino-3-hydroxy-5-methyl-4-isoxazolepropionic acid receptor (AMPAR), the γ-aminobutyric acid receptor-B (GABA_B_ receptor), and voltage-gated potassium channels (VGKCs) [1]. VGKCs are now known to be leucine-rich glioma-inactivated protein 1 (LGI1) and contactin-associated protein-like 2 (Caspr2) [2]. Since antibody LGI1 encephalitis patients are characterized by acute or subacute onset of cognitive dysfunction, the disease has often been misdiagnosed as a mental illness in the past. Recent studies have also deepened the understanding of LGI1 antibody encephalitis. For example, Shin et al. indicated that this disease accounted for 11.2% of all autoimmune encephalitis [10]. Results from European-based studies showed that the peak age of disease onset was between 61 and 64 years and that males accounted for 55–66% of the patient population, while the annual rate of incidence was 0.63–0.83/million [11, 12]. The average age of onset of the disease of the eight cases in our study was 63.4 years old, and five were male, which was in line with the European data.

Our study also showed that epilepsy and cognitive impairments were common and prominent clinical manifestations in patients with LGI1 antibody encephalitis. Epilepsy was the initial symptom in the majority of the patients, with several patients exhibiting multiple forms of seizures. Seizures involving ipsilateral face and/or limb dystonia-like seizures (FBDS) were the most common characteristic. We also showed FBDS were short duration and high in frequency. In most cases, the seizures were not accompanied by conscious disturbances, and in several instances, they were difficult to capture by EEG. These clinical features agree with previous reports [2, 11]; FBDS appear earlier than other symptoms in many patients [10, 13], while tonic-clonic seizures often occur concurrently or immediately after a decline in patient cognitive function [11]. Therefore, it was suggested that following initial FBDS, immediate immunotherapy treatment might prevent the development of cognitive impairments [14]. In our study, six patients had FBDS, and four patients experienced these seizures prior to cognitive impairment. Interestingly, the conditions of these patients gradually worsened. We believe this was a direct result of being administered only antiepileptic treatments and not immunotherapy treatments. Given that FBDS occur early and have a high incidence in LGI1 antibody encephalitis, we suggest that all patients with FBDS be examined for increased LGI1 antibody titers as soon as possible, and upon definitive diagnosis, immunotherapy be initiated immediately. These measures may largely prevent the onset of cognitive impairment.

Interestingly, it is still arguable whether FBDS are dystonic or epileptic seizures. Epileptic waves have been recorded in patients during FBDS [15], while studies also showed that epileptic seizures occurred if LGI1 gene expression was deficient [6, 16]. However, others believe that the causative lesions in patients with FBDS occur in the basal ganglia and do not affect EEG readings, and therefore, FBDS is a type of dystonia derived from deep brain dysfunction [17, 18]. In our study, two patients had FBDS-associated basal ganglia lesions, suggesting that FBDS may be associated specifically with these lesions. Therefore, the specific mechanisms involving the onset of FBDS need further study.

Our results regarding memory deficits and disorientation as the primary manifestations of LGI1 antibody encephalitis-associated cognitive impairment in the patients are in agreement with previous studies, as were results demonstrating changes in both personality and mood and patient hallucinations (visual and/or auditory) [11, 12, 19]. It has been proposed that memory impairment is due to the interaction of LGI1-ADAM22-AMPAR affecting long-term depression and long-term potentiation [20]. Collingridge et al. found that long-term depression was also closely associated with the formation of spatial memory [21]. In our study, two patients were first treated for dementia because in both cases, their initial symptom was memory deficit. Therefore, caution is needed when making initial diagnoses in middle-aged and elderly patients who present with memory deficit. In addition to the aforementioned typical symptoms, patients with LGI1 antibody encephalitis can also exhibit, among other symptoms, sleep or autonomic disorders and ataxias [11, 14, 22, 23]. Although less frequent, these symptoms also occurred in the patients in our study.

Over half of patients suffering from LGI1 antibody encephalitis also exhibit hyponatremia and in most cases, refractory hyponatremia [2, 12]. The pathogenic mechanism involved in the onset of hyponatremia is likely associated with lowered antidiuretic hormone levels due to the effects of LGI1 antibodies on the hypothalamic paraventricular nucleus and kidney [24]. In our study, hyponatremia occurred in six patients, and three presented with refractory hyponatremia, highlighting the prevalence of hyponatremia in patients with LGI1 antibody encephalitis.

Patients with LGI1 antibody encephalitis are often normal in routine CSF tests [25]; the positive rate of LGI1 antibody detection in CSF is lower than that in serum [26]. Even if CSF is positive for LGI1 antibodies, its titer is only 1 to 10% of a serum titer [27]. Therefore, if LGI1 antibody encephalitis is suspected, routine serum tests for autoimmune encephalitis antibodies are first performed, thus occasionally negating the need for invasive lumbar punctures. As expected, both serum and CSF LGI1 antibody titers can decrease or increase with the disease remission and relapse, respectively [3, 28]. However, Ariño et al. showed that lowered LGI1 antibody titers were not significantly associated with patient prognosis [26], and in agreement with this, one patient in our study who recovered did not have decreased LGI1 titer levels compared with disease onset levels. Taking these findings together, we speculate that antibody titer does not always correlate with disease severity and therefore needs further investigation.

Approximately 70% of patients with LGI1 antibody encephalitis have increased T2 and FLAIR MRI signals in the hippocampus or temporal lobe (unilaterally or bilaterally), and some can extend to the amygdala, insula or striatum [11, 12]. Flanagan et al. found that ~ 40% of patients also had basal ganglia lesions corresponding to FBDS, and T1 hyperintensities either occurred concurrently with or were preceded by short-lived T2 hyperintensities during the episodes, with T1 hyperintensities persisting longer than T2 hyperintensities; regarding the T1 hyperintensity pathophysiology, the authors suggest that hypoxic damage is the most likely substrate [29]. Examples of these abnormal images can be found in our study: the follow-up MRIs of 5 months showed that basal ganglia lesions completely regressed in one patient, although the condition of the patient was exacerbated (Fig. 1 E1-E2); another patient showed increased T1 signaling in the initial lesion region after 1and 2 months later (Fig. 1 D1-D3). The above results suggest that the presence or absence of abnormal MRI findings is related to the time of onset. The different MRI examination times may lead to the illusion that the imaging results and clinical symptoms are inconsistent. Therefore, the time when the T1 and T2 abnormalities occur and the time of existence requires further study.

In view of similar clinical manifestations, the diagnosis of LGI1 antibody encephalitis need to be distinguished from viral encephalitis, Hashimoto’s encephalopathy, Creutzfeldt’s disease (CJD), and other forms of autoimmune encephalitis [30]. In combination with clinical manifestations, laboratory tests, and imaging examinations, the diagnosis of LGI1 antibody encephalitis is usually correct; however, it should be noted that increased LGI1 antibody titers were also found in a pathologically confirmed CJD case [31]. We therefore recommend using a combination of examinations to confidently and correctly diagnose LGI1 antibody encephalitis.

First-line therapies for the disease include intravenous glucocorticoid therapy and immunoglobulin and plasma exchange, with early combinatorial treatments providing better efficacy [10, 11, 30]. In addition to these combinatorial treatments, it is sometimes necessary to supplement treatment with cyclophosphamide or rituximab [32]. Studies have shown that ~ 80% of patients had a reduction in seizures and had improvements in cognitive impairments after a 2-week first-line treatment regimen; while 70% of patients had a good prognosis after a 2-year follow up, the recurrence rate was ~ 30%, and most recurrences occurred in the first 6 months; a small number of patients needed long-term administration of oral immunomodulatory agents and AEDs; and finally, the mortality rate of LGI1 antibody encephalitis is 6–19% [11, 26].

In the present study, all eight patients showed symptom improvement after a 2-week first-line treatment regimen. Patients were followed up for 4–13 months, and the overall recovery was good. The major remaining symptoms were amnesia, spatial disorientation, and insomnia. Two patients relapsed within 3 months and presented with frequent episodes of FBDS, which were reversed after intravenous injection of glucocorticoids and immunoglobulins. In the final follow-up, seven patients still took oral AEDs, and only one patient had occasional seizures.

### Limitations

We obtained the most reliable data by analyzing patients’ files and interviewing patients, relatives, and treating physicians, and all MRIs were reviewed by a specialized neuroradiologist. However, given the retrospective nature of this study, the limited number of patients, the short duration of the follow-up, and the variable laboratory and brain MRI timing, the conclusions about clinical characteristics and the short-term prognosis of LGI1 antibody encephalitis have limitations.

## Conclusion

The primary symptoms of LGI1 antibody encephalitis include cognitive impairment (recent memory loss or spatial disorders), seizures (typically FBDS), hyponatremia, and sleep disorders, while serum titers (and occasionally cerebrospinal fluid) of LGI1 antibodies are increased. In addition, brain MRIs may indicate abnormal signals in the temporal lobe, hippocampus, or basal ganglia.

Early immunotherapy can achieve both increased efficacy and good long-term prognosis. We believe that if patients present with recurrent epileptic seizures and cognitive dysfunctions, LGI1 antibody testing should be promptly performed and appropriate treatments given immediately. These measures will not only improve seizure control but also may improve long-term prognosis.

## Abbreviations

AEDs: Antiepileptic drugs
AMPAR: α-amino-3-hydroxy-5-methyl-4-isoxazolepropionic acid receptor
Caspr2: Contactin-associated protein-like 2
CJD: Creutzfeldt’s disease
CSF: Cerebrospinal fluid
EEG: Electroencephalography
FBDS: Faciobrachial dystonic seizure
FLAIR: fluid-attenuated inversion recovery
GABA-B receptor: γ-aminobutyric acid receptor-B
LGI1: leucine-rich glioma-inactivated 1
MRI: magnetic resonance imaging
NMDAR: N-methyl-D-aspartate receptor
VGKC: Voltage-gated potassium channel complex
VGKCs: Voltage-gated potassium channels
